# The impact of two radical sternectomy surgical techniques on the outcome of deep sternal wound infections

**DOI:** 10.1186/s13019-024-02491-7

**Published:** 2024-01-24

**Authors:** Olimpiu Bota, Feras Taqatqeh, Florian Bönke, Maxime Mülhausen, Klaus Matschke, Adrian Dragu, Kevin Bienger, Stefan Rasche

**Affiliations:** 1 Faculty of Medicine Carl Gustav Carus, University Center for Orthopedics, Trauma and Plastic Surgery, TU Dresden, Fetscherstraße 74, 01307 Dresden, Germany; 2 Department of Cardiac Surgery, University Heart Center Dresden, TU Dresden, Fetscherstrasse 76, 01307 Dresden, Germany; 3 Surgical Intensive Care Unit, Faculty of Medicine Carl Gustav Carus, TU Dresden, Fetscherstraße 74, 01307 Dresden, Germany

**Keywords:** Deep sternal wound infection, Radical sternectomy, Sternal reconstruction, Cardiac surgery

## Abstract

**Background:**

Deep sternal wound infection (DSWI) is a rare, yet devastating complication after cardiac surgery. While the surgical treatment always implies the soft tissue and bone debridement, there is little data about this procedure. The aim of our study was to evaluate the impact of the radical sternectomy on the outcome in patients with DSWI and to identify the risk factors which could influence the result. The surgical techniques of piecemeal sternectomy and the newly developed en bloc sternectomy were also evaluated.

**Methods:**

The study was developed as a retrospective cohort study. 86 patients with DSWI who received a radical sternal resection at our institution between March 2018 and December 2021 were included.

**Results:**

The average age of the cohort was 67.3 ± 7.4 years, and 23.3% of patients were female. The average length of stay trended shorter after en bloc sternectomy (median 26 days) compared to piecemeal sternectomy (37 days). There were no significant differences between the piecemeal and en bloc sternal resection techniques. Anticoagulant and antiplatelet drugs had no significant influence on bleeding and transfusion rates. Obese patients showed an increased risk for postoperative bleeding requiring reintervention. Transfusion of packed red blood cells was significantly associated with lower hemoglobin values before surgery and ASA Class 4 compared to ASA Class 3. The in-hospital mortality was 9.3%, with female sex and reintervention for bleeding as significant risk factors. Nine patients developed an infection relapse as a chronic fistula at the level of clavicula or ribs, with ASA Class 4 as a risk factor.

**Conclusion:**

Radical sternectomy is a safe procedure to treat DSWI with compromised sternal bone. Both piecemeal and en bloc techniques ensure reliable results, while complications and mortality appear to be patient-related.

**Supplementary Information:**

The online version contains supplementary material available at 10.1186/s13019-024-02491-7.

## Background

One of the most feared complications after median sternotomy is the development of a deep sternal wound infection (DSWI) [1]. This complication implies an infection that not only encompasses the skin and the subcutaneous tissues, but also the underlying bonce and the mediastinal tissues. The incidence of DSWI lies around 1.6% [2]. The developments in the treatment of this condition, especially the introduction of negative pressure wound therapy (NPWT) have remarkably reduced the mortality from 50% to around 10% nowadays [1, 3]. Several factors have been incriminated to facilitate the development of DSWI, which can be related to comorbidities, surgical technique and hospital stay. Out of these, especially the bacterial contamination of the surgical wound as well as the devascularization of the sternum due to the harvest of one or both internal mammary arteries lead to the development of osteomyelitis [4]. In incipient cases, the infected bone can be debrided and an attempt to rewire the sternum can be made, if enough bone substance is left. In advanced cases, where the complete sternum is infected or necrotic or when there is not enough bone substance left to rewire the sternum, a radical debridement of the sternum is needed.

The concept of treatment for sternal osteomyelitis (SO) has shifted over the years from a restrictive treatment to a more radical method. After a radical debridement of the sternum usually the wound is conditioned using negative wound pressure therapy (NPWT) [5]. Finally, the sternal reconstruction is performed using pedicled or free flaps. The latissimus dorsi musculocutaneous flap (LDMF) and the pectoralis major flap remain two of the most reliable sources of soft tissue [6, 7]. The success of therapy relies on prompt, standardized treatment in an interdisciplinary setting.

Although the radical removement of infected or necrotic tissue is of paramount importance for the successful treatment of DSWI, there is little data in the literature on sternal debridement. With most surgical publications concentrating on sternal coverage. The primary goal of this work was to evaluate the impact of the radical sternectomy on the patient’s course. Secondarily we aimed at comparing the already established piecemeal resection of the diseased sternal bone with the newly developed en bloc sternectomy and to identify other factors which could influence the outcome. Thirdly we aimed at showing that the intake of antiplatelet and anticoagulant agents does not increase mortality.

## Material and methods

Between March 2018 and December 2021, 86 patients with DSWI and SO after primary cardiac surgery were treated with radical sternal resection and subsequent flap reconstruction. The study was approved by the ethics committee of our institution (registration number EK 387082020 from 10/21/2020) and was developed as a retrospective cohort study and performed in cooperation between the Departments of Plastic Surgery and Cardiac Surgery, which performs around 2,200 open heart surgeries a year. The patients provided informed consent for the publication of the clinical photos.

**Fig. 1:**
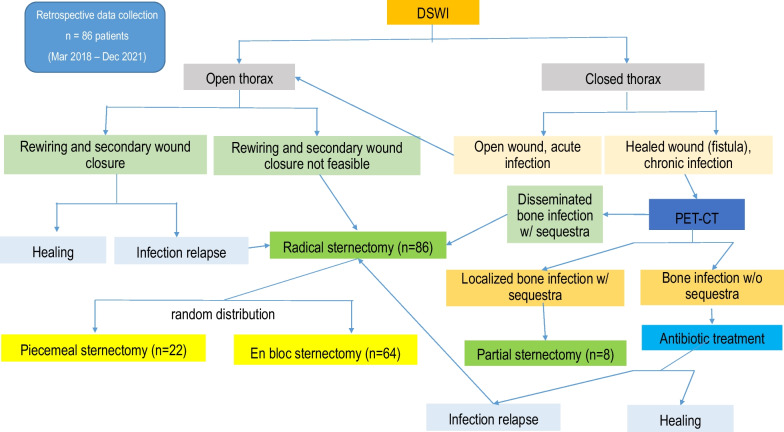
Flowchart depicting the patient selection criteria according to STROBE

**Fig. 2:**
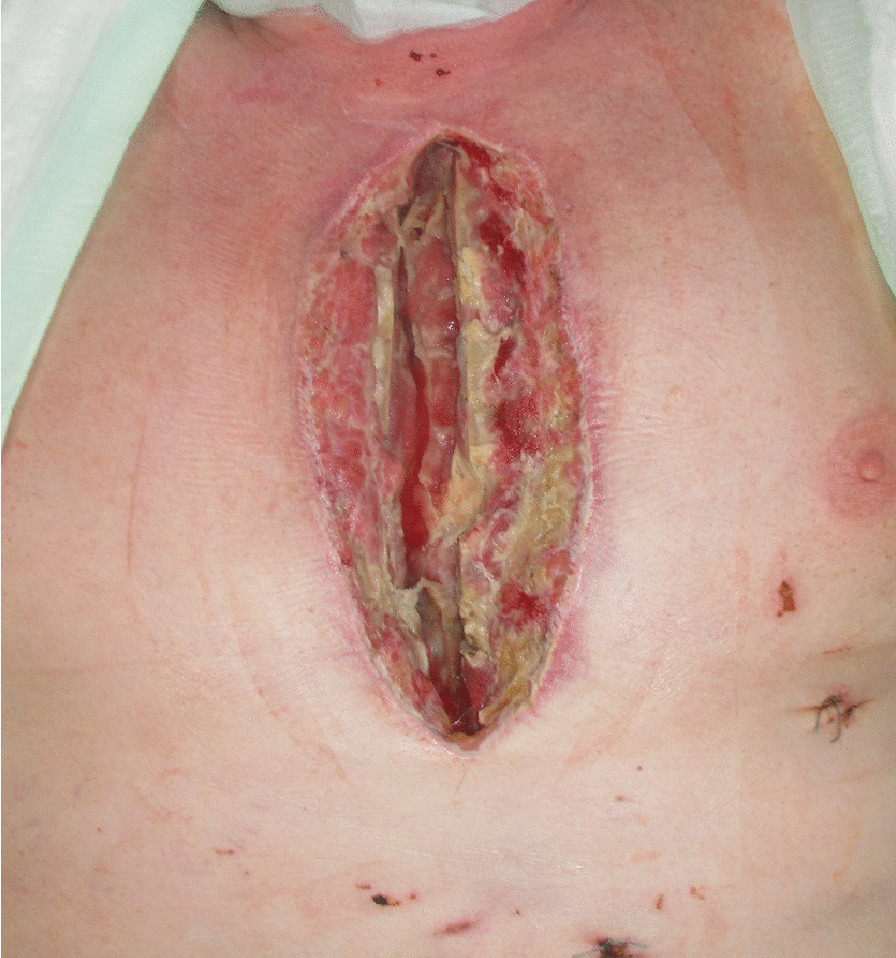
Typical clinical image of a DSWI before debridement

**Fig. 3:**
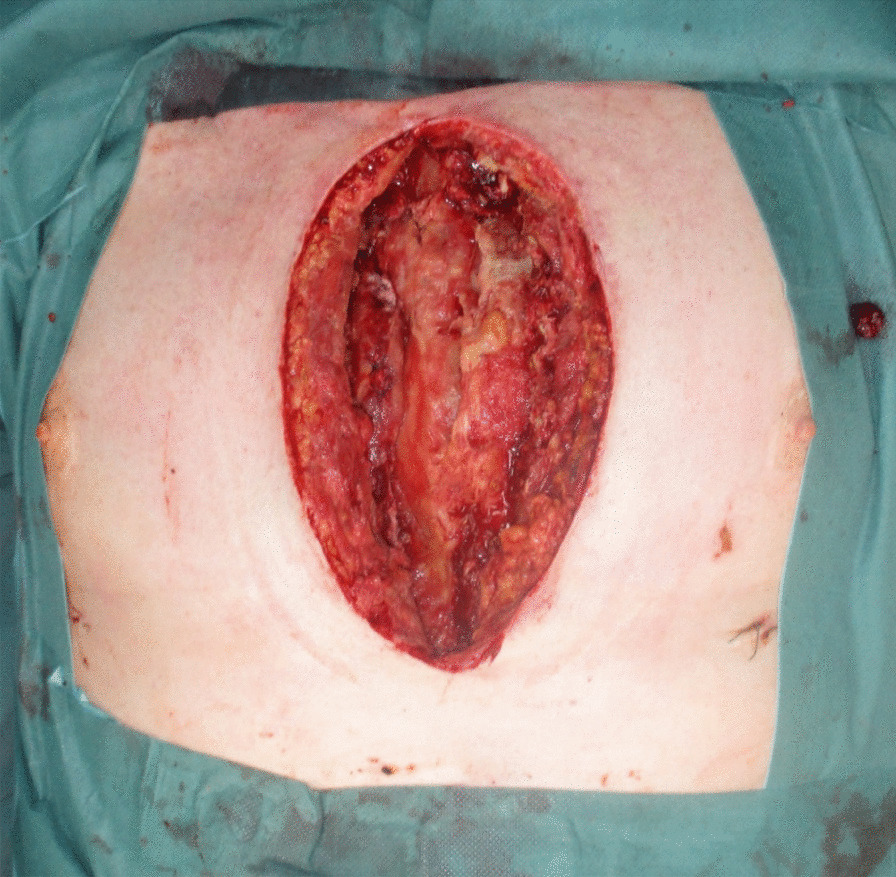
Deep sternal wound after radical sternal resection

**Fig. 4:**
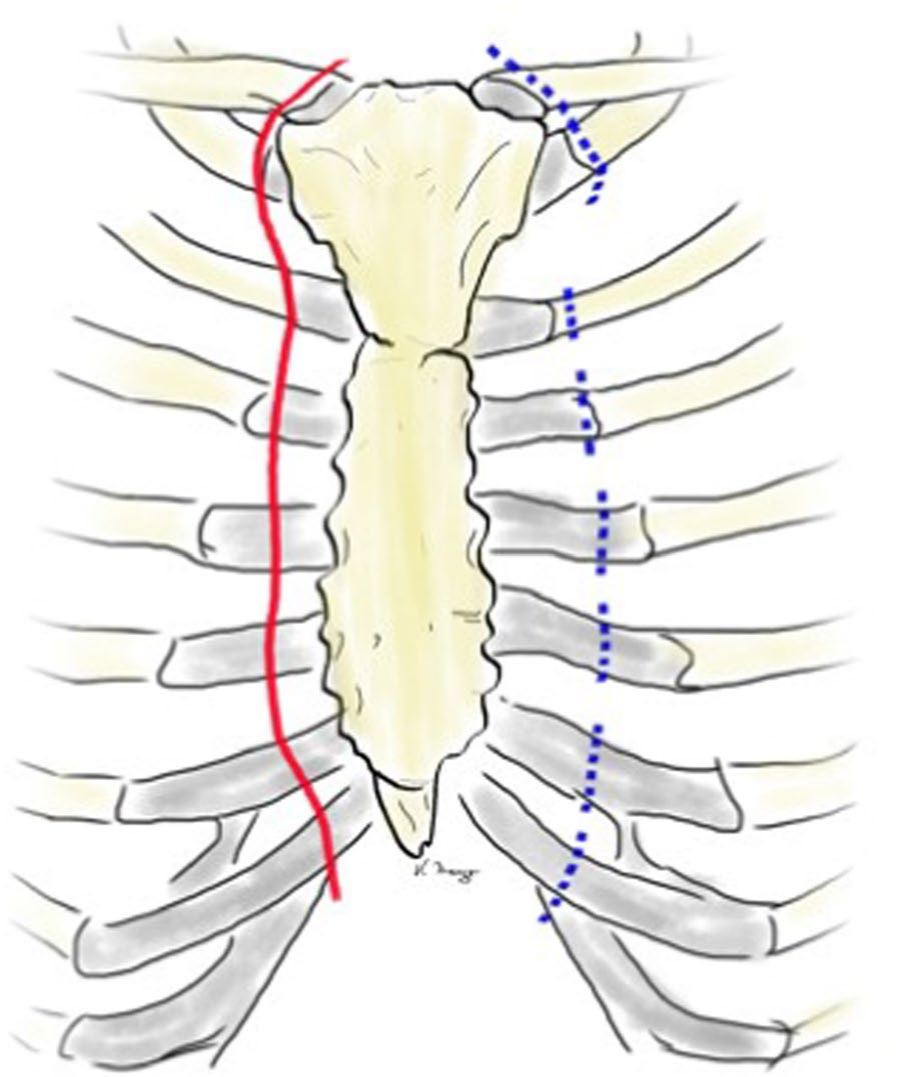
Schematic illustration of the level of sternal resection (red line) and rib resection (blue line)

The diagnosis of DSWI was mainly established in the Department of Cardiac Surgery and included the CDC (Centers for Diesease Control and Prevention) criteria: isolation of a microorganism from the mediastinal culture or intraoperative evidence of mediastinitis or the presence of sternal instability, pain, fever > 38°C and purulent drainage from the sternal wound [8]. The therapeutical standard includes wound revision, local debridement and NPWT. After treatment of the local infection, sternal rewiring with a secondary wound closure is favored. In the cases where a secondary wound closure was not possible because a high suspicion of SO, destruction of bone structure or when the infection relapsed after secondary closure, the patient was transferred to the Department of Plastic Surgery, where a standardized treatment was established (Fig. 1). In patients with an open sternal wound, the indication for sternal resection was made based on the clinical, microbiological and histopathological evaluations. When the thorax was closed, especially in chronic DSWI, positron emission tomography/computed tomography (PET/CT) was performed, to the extent of osteomyelitis and bone destruction. In localized infections, only a partial sternal resection (in the horizontal plane) with complete wire removal was performed, leaving a part of the thoracic circle in continuity. These patients were excluded from this study, which only examines the patients with radical sternectomy (Fig. 1). Infections that first occurred or relapsed in the first three months after the median sternotomy were defined as acute, whereas infections that occurred after three months were defined as chronic. Treatment consisted in the radical debridement of the necrotic soft tissues as well as a complete removal of the suspected infected sternal bone in terms of a sternectomy (Figs. 2, 3, 4). This took place either in a classical manner as a piecemeal resection using a rongeur or in a newly developed technique, with an en bloc resection of each hemisternum or the whole sternum, as described beneath. The minimum follow-up time was six months.

### Operative technique

With the patient in the supine position, the arms are abducted 90°. The anterior thorax, including the neck and the upper abdomen are prepped and draped (Fig. 3). One inguinal region is also separately draped, for any emergency case, in which a heart–lung machine needs to be installed. The surgery begins by excising the scar tissue, the fistulas or the reepithelized wound margins, if the wound has already been opened, while minimizing the wound extension cranially and distally. If the sternal wound stretches too far cranially, the placement of a future tracheostomy may be jeopardized and if the wound extends too far distally, the abdominal cavity may inadvertently be opened.

**Fig. 5:**
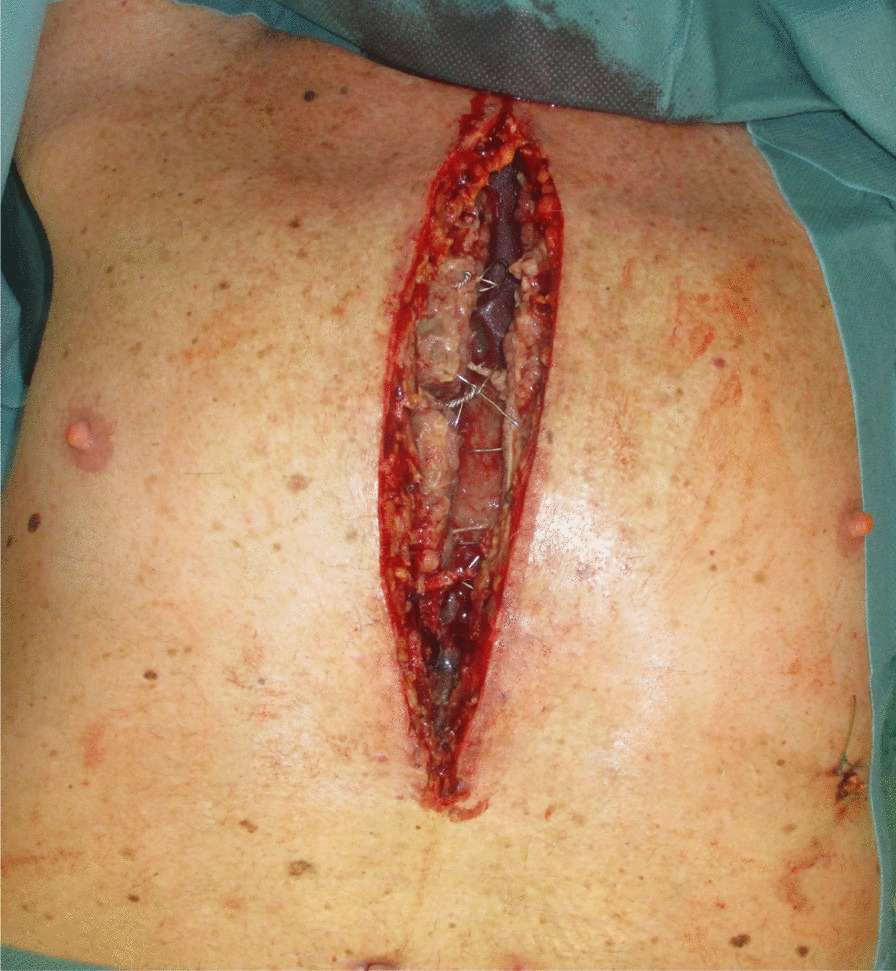
Deep sternal wound with sternal dehiscence, broken wires and fractured bone

The electrocautery dissection continues afterward straight to the sternum and the soft tissues are elevated laterally, until the rib cartilages and the sternoclavicular joints are freed. Downwards the dissection stops at the level of the xiphoid, while upwards the dissection stops at the lower neck. At this point the sternal wires, if still in situ, are being removed and the two sternal sides are split apart (Fig. 5). Using periosteal elevators and scalpel, the sternum is elevated from the dorsal periosteum (Additional file 2: Fig. S1). The dissection may begin on the left side of the patient received a left internal mammary bypass, as the dissection may be easier here. Care must be taken not to injure the pericard or the lungs, which may be adherent to the sternum after previous surgeries. After freeing the sternum laterally to the rib cartilages, to the first rib and the sternoclavicular joints (Fig. 4), a large Ruskin Liston bone cutting forceps is used to sever the ribs from caudal to cranial (Additional file 2: Fig. S2). Finally, the sternoclavicular joint is opened (Additional file 2: Fig. S3) and the hemi-sternum is excised and sent to histological examination. If the internal mammary arteries (if still in place) are injured, they can be safely ligated. Pleural or lung fistulas can be sealed with a sponge sealant patch or directly sutured.

Next, the necrotic mediastinal tissues are carefully resected. The remaining rib cartilages are removed using a rongeur, as well as the articular disc of the sternoclavicular joint. Finally, the wound is sealed with NWPT with instillation. A three-point compression girdle is applied to the chest to stabilize the thorax and is worn until six weeks after flap closure.

If the two hemisternums are united through fibrous or bony tissue but the PET/CT shows a disseminated osteomyelitis with sequestra, the sternectomy is performed starting from the lower edge of the sternum and ribs and proceeding cranially, while detaching the bone dorsally from the mediastinum and severing the ribs on both sides (Additional file 2: Fig. S4). Finally, the sternoclavicular joints are opened and the whole sternum is resected (Additional file 2: Fig. S5, Additional file 3: Video S1).

### Data analysis and statistics

Data are reported as frequencies and percentages for categorical data and as mean and standard deviation (sd) or median and interquartile range (IQR) for continuous data, depending on their distribution. Normal distribution was tested with the Shapiro–Wilk test. Categorial data were compared by Fisher´s Exact test, continuous data with the t-Test if normally distributed or with the Mann–Whitney-U-test otherwise. Non-significant outcome differences between the two techniques were subsequently tested for equivalence by a “two one-sided tests procedure” (TOST)[9] and followed by power analyses for equivalence or difference, depending on the TOST result. Uni- and multivariable linear or logistic regression was applied for continuous or binary outcome variables, respectively. Multivariable models were constructed with selected variables in question and pared down based on stepwise deletion. A proportional odds logistic regression was employed for the outcome of transfusion requirements, which is given in four categories. Power analyses and sample size calculations were performed[10]. Statistical significance was set at p < 0.05. Statistics were calculated with R, Version 3.6.1 (R Foundation for Statistical Computing: Vienna, Austria, 2019) [11].

## Results

A total of 94 patients with DSWI were treated during the period studied, of which 86 patients (20 women [23.3%] and 66 men [76.7%]) were included. The mean age of the patients at the time of surgery was 67.3 ± 7.4 years.

Eight patients who did not receive a complete sternal resection were excluded from the study. These patients had a united sternum with either chronic manubrial osteomyelitis, without the involvement of the corpus sterni, or they showed a stable sternum and the therapy proceeded with prolonged antibiotic treatment. Out of the 86 included patients, 22 received a piecemeal sternal resection (PMSR), while the other 64 patients received an en bloc sternal resection (EBSR). There were 82 acute DSWI and four chronic (longer than three months since median sternotomy). No statistically significant differences were noted between the two groups concerning sex, age, American Society of Anesthesiologists physical status (ASA), or the number of obese patients with a Body Mass Index (BMI) ≥ 30 kg/m^2^ based on the definition of the World Health Organisation[12] (Table 1). Furthermore, there was no statistically significant difference concerning the intake of antiplatelet or anticoagulant drugs between the two groups as well as for the existence of a coronary bypass using the left internal mammary artery (LIMA), which was routinely harvested as skeletonized. The harvest of both internal mammary arteries was only encountered in one patient.

**Table 1:** Baseline characteristics, type of preceding surgery and anticoagulation/platelet inhibition

	All	PMSR	EBSR	p
Number of patients	86	22	64	
Age (years)	67.3 ± 7.4	65.9 ± 7.9	67.8 ± 7.3	0.315
Sex, n (%)				0.575
Male	66 (76.7)	16 (72.7)	50 (78.1)	
Female	20 (23.3)	6 (27.3)	14 (21.9)	
ASA class, n (%)				0.75
ASA III	71 (82.6)	19 (86.4)	52 (81.3)	
ASA IV	15 (17.4)	3 (13.6)	12 (18.7)	
NYHA class				0.485
NYHA 1	3 (3.8)	1 (5.0)	2 (3.4)	
NYHA 2	26 (32.9)	5 (25.0)	21 (35.6)	
NYHA 3	46 (58.2)	14 (70.0)	32 (54.2)	
NYHA 4	4 (5.1)	—	4 (6.8)	
Diabetes mellitus, n (%)	54 (62.8)	13 (59.1)	41 (64.1)	0.799
On insulin, n (%)	25 (29.1)	7 (31.8)	18 (28.1)	0.789
Atrial fibrillation, n (%)	31 (36.0)	8 (36.4)	23 (35.9)	1
COPD, n (%)	17 (19.8)	3 (13.6)	14 (21.9)	0.541
BMI^1^				0.629
Non-obese, n (%)	47 (54.7)	11 (50.0)	36 (56.3)	
Obese, n (%)	39 (45.3)	11 (50.0)	28 (43.8)	
Platelet inhibitor, n (%)				0.901
None	12 (14.0)	3 (13.6)	9 (14.1)	
Singe	68 (79.1)	17 (77.3)	51 (79.7)	
Double	6 (7.0)	2 (9.1)	4 (6.3)	
Therap. heparine, n (%)	52 (60.5)	16 (72.7)	36 (56.3)	0.212
preceding cardiac surgery				
CABG	43 (50.0)	18 (81.8)	25 (39.1)	0.001
LIMA^2^	61 (70.9)	15 (68.2)	46 (71.9)	0.789
Valve	11 (45.3)	2 (9.1)	9 (14.6)	0.721
CABG + Valve	23 (26.7)	—	23 (35.9)	0.0001
Aortic	8 (9.3)	2 (9.1)	6 (9.4)	1
Non-cardiac^3^	1 (1.1)	–	1	–

Values are presented as mean ± standard deviation or n (%). P-values indicate the statistical difference between sternal resection techniques

PMSR, piecemeal sternal resection; EBSR, en bloc sternal resection. BMI, body mass index, ASA Class, American Society of Anesthesiologists physical status classification; NYHA class, New York Heart Association functional classification; COPD, chronic obstructive pulmonary disease; CABG, isolated coronary artery bypass graft; LIMA, use of the left internal mammary artery as bypass craft; Valve, isolated valvular surgery. Aortic surgery comprises isolated aortic conduit (n = 2) and combined coronary or valvular procedures (n = 6))

^1^Based on the WHO Definition of obesity at a body mass index >  = 30 kg/m^2^

^2^Sum of isolated CABG and combined procedures

^3^Sternal resection for sarcoma

The intake of antiplatelet drugs (acetylsalicylic acid and Clopidogrel) and anticoagulants (heparin and low molecular weight heparin) is common in patients after cardiac surgery. 74 patients in our cohort received at least one of the above-mentioned drugs (Table 1). The PMSR and EBSR groups had an equal distribution concerning the intake of anticoagulant and antiplatelet drugs.

The median length of hospital stay was tendentially shorter in the EBSR than in the PMSR group (26 versus 37 days, p = 0.051). Multivariable linear regression confirmed the narrow non-significance for the effect of the resection technique on the length of hospital stay (p = 0.063) but showed a shorter treatment for women than men (p = 0.008), and prolonged treatment of those patients who required more than 4 transfusions of packed red blood cells after surgery (p = 0.009), or who developed a fistula after surgery (0.002). Concerning the duration of surgery, the PMSR lasted 61.5 (IQR 54.3 – 65.8) minutes, while the EBSR lasted 69 (IQR 55.3—90.3) minutes on average (p = 0.12, Mann–Whitney Test). The intraoperative and immediate complications were also evaluated. While there were no aggravating intraoperative events, the lesion of the parietal pleura, the lung parenchyma or the pericard may occasionally occur due to the postoperative and inflammatory adhesions. The opening of the parietal pleura occurred in one case in the PMSR group and in 22 cases in the EBSR group (p = 0.005, Fischer’s exact test). An injury of the lung parenchyma was observed in 2 cases in the PMSR group and in 7 cases in the EBSR group (p = 1). In two PMSR cases and in six EBSR cases the pericardium had to be opened during debridement.

There was no persistent pneumothorax recorded in the period between the sternectomy and the flap coverage. The most relevant postoperative incident was the occurrence of relevant bleeding. There were 2 cases in the PMSR group and 6 cases in the EBSR group which had to be brought back to the operating room to control the bleeding, all in male patients. There was no difference in the reintervention rate for bleeding between resection techniques (p = 1.0, Fisher´s Exact Test). However, the equivalence of the reintervention rate in PMSR and EBSR could not be confirmed (p = 0.08). To verify the equivalence of relevant bleeding rates between groups within a 10% boundary with a power of 0.8, 153 patients per group would have had to be studied. In the univariable analysis, there was no statistical association between the reintervention for bleeding and the intake of anticoagulant and antiplatelet drugs, sex, age, ASA physical status, LIMA, prothrombin time before sternectomy or the resection technique. Partial thromboplastin time was markedly increased (59 s) in one of the eight patients with reintervention for bleeding but had otherwise no statistical association with that outcome. Obese patients developed bleeding more often (p = 0.035). The multivariable adjustment did not affect this result, leaving obesity as the only risk factor (Additional file 1: Table S1).

Most postoperative bleedings can usually be controlled without a surgical reintervention. Adjusting the coagulation parameters, administration of antifibrinolytic drugs (Tranexamic Acid), local compression, pausing the NPWT for a short period or temporary removal of the NPWT and packing usually controls the bleeding. We analyzed the preoperative value of hemoglobin, as well as the administration of red cell concentrates during and after the sternectomy, up to the flap coverage. Transfusion requirements were categorized into four groups, none, 1–2 units of RBC, 3–4 units of RBC or more than 4 units of RBC. Univariable and multivariable analyses showed that the resection technique, obesity, intake of heparin products or antiaggregant agents, prothrombin time, LIMA bypass, sex and age did not affect postoperative transfusion. The likelihood to be in a higher transfusion category was only related to the preoperative hemoglobin value and the ASA status, whereas the PTT before surgery had a marginally nonsignificant impact (p = 0.06, Additional file 1: Table S2). ASA class 4 increased this probability 3.5-fold, compared to ASA class 3.

The in-hospital mortality in the entire collective was 9.3%. Three of 22 patients (13.6%) died after PMSR and five of 64 patients (7.8%) after EBSR (p = 0.70). Although the difference in death rates between both resection techniques was not statistically significant, their equivalence could not be confirmed. Within a 10% boundary, the lower limit of the proportion difference *p*_[PMSR]_-*p*_[EBSR]_ was verified (p = 0.025), but the upper limit was rejected (p = 0.302). Thus, a more than 10% lower mortality after piecemeal sternal resection is unlikely, but a more than 10% lower mortality after en bloc resections cannot be ruled out. To confirm such a difference with a power of 0.8, 343 patients in each group would need to be studied. Relating comorbidity and patient characteristics, only diabetes mellitus, which required treatment with insulin, affected mortality. Five of 25 patients (20%) treated with insulin died, compared to three of 61 patients (4.9%) not on insulin treatment (p = 0.043). The higher mortality of 20.0% in women compared with 6.1% in men, was marginally nonsignificant (p = 0.08). The type of resection, obesity, age, partial prothrombin time, ASA physical status, NYHA functional status, the opening of the pleural cavity, lesion of the lung parenchyma or the preoperative hemoglobin value and the intake of heparin products did not influence mortality. Bleeding requiring reintervention did not affect mortality in the whole cohort but occurred only in men. Adjusting for the effect of bleeding by gender in a bivariate logistic regression model identified a marginal non-significant effect on mortality (odds ratio 9.3, 95% CI 0.98 to 90.7, p = 0.04). Of note, the higher mortality in women became significant after the adjustment (odds ratio 7.0, 95% CI 1.25 to 53.9, p = 0.033).

Power and sample size were calculated for significant results. A sufficient test power (0.82) was demonstrated for the effect of obesity on bleeding requiring reintervention. The effects of insulin therapy, gender and significant bleeding on mortality and that of ASA physical status on chronic fistula did not meet the statistical power of 0.8 at the given sample sizes (Additional file 1: Table S2).

All resected sternums in both groups showed signs of osteomyelitis at the histological examination. The histopathological examination of the anatomical parts of the hemisternums will be published in a subsequent paper. The identified pathogens were Coagulase-negative Staphylococci, Staphylococcus aureus and gram-negative bacteria in even proportions. A detailed microbiological study of all 94 patients will also be subsequently published.

The follow-up time was on average 94 days (30–410). To evaluate the efficiency of the sternal debridement and infection treatment, the occurrence of a chronic cutaneous fistula from the remaining bones (medial clavicula or medial rib stumps) was assessed (Table 2). All these patients were surgically treated with resection of the infected bone (clavicula or rib), soft tissue sanitation using NPWT and secondary wound closure. ASA 4 patients showed an increased risk of developing chronic cutaneous fistula compared to ASA 3 patients (OR 4.8, 95 CI 1.1 to 21.1, p = 0.03), while the type of resection, age, sex, obesity, the intake of antiplatelet and anticoagulant agents, partial thromboplastin time, prothrombin time and relevant postoperative bleeding did not influence the infection recurrence.

**Table 2:** Outcomes after sternal resection. Values are presented as median (interquartile range) or n (%). p-values indicate the statistical difference between sternal resection techniques

	All	PMSR	EBSR	
Number of patients	86	22	64	
Hospital stay (days, median (IQR))	28 (21–39)	37 (26–42)	26 (21–34)	0.051
Duration of surgery (min, median (IQR))	65 (54.3—72.4)	61.5 (54.3—65.8)	69.0 (55.3—90.3)	0.123
Pleural opening, n (%)	23 (26.7)	1 (4.5)	22 (34.4)	0.005
Fistula, n (%)	9 (10.5)	2 (9.1)	7 (10.9)	1
Pericardial adhesions, n (%)	8 (9.3)	2 (9.1)	6 (9.4)	1
Packing or redo for bleeding, n (%)	8 (9.3)	2 (9.1)	6 (9.4)	1
Packed RBC, n (%)				0.633
0 units	20 (23.3)	7 (31.8)	13 (20.3)	
1–2 units	25 (29.1)	7 (31.8)	18 (28.1)	
3–4 units	24 (27.9)	5 (22.7)	19 (29.7)	
> 4 units	17 (19.7)	3 (13.6)	14 (21.9)	
Mortality, n (%)	8 (9.3)	3 (13.6)	5 (7.8)	0.416

PMSR, piecemeal sternal resection; EBSR, en bloc sternal resection; RBC, red blood cells

Olimpiu Bota is a plastic surgeon, currently assistant medical director at the Center of Orthopedics, Trauma and Plastic Surgery, specialized in reconstructive surgery, with a broad experience in the treatment of deep sternal wound infections. He is a member of the German societies of plastic surgery, hand surgery and surgery

## Discussion

In the current study, we evaluated the impact of radical sternectomy in the treatment of DSWI and showed that PMSR as well as EBSR had similar results in efficiently treating the infection with similar mortality and infection recurrence rates.

The surgical infection treatment classically involves the removal of all non-viable tissues to reduce the bacterial burden and facilitate healing [13]. In DSWI the sternal bone is often affected by the infection, leading to SO. Nevertheless, the single existence of bone infection does not preclude the possibility of wound healing, as local treatment with irrigation, NPWT and prolonged systemic antibiotic therapy might lead to wound closure [14]. The threshold at which the sternal bone must be resected lies in our opinion at the level of bone destruction and bone loss. In cases where the sternum has been progressively narrowed through multiple wound revisions and closure with rewiring or plating would not be possible or when there are multiple transverse fractures due to wire infraction and infection, a complete sternal removal is inevitable. Time also plays a crucial role in the decision-making progress, as DSWI patients are multimorbid and often hospitalized. The unnecessary prolongation of treatment comes at a cost for the patient and boosts morbidity, mortality and costs [2, 15–17].

DSWI can occur months after the open-heart surgery, when the skin wound has healed and the sternal bone may show signs of fibrous or bony union. As an acute intervention is usually not indicated in these cases, radiologic diagnosis is useful before treatment. PET/CT has been proven to have a high sensitivity, specificity and accuracy in detecting the extent of DSWI not only in-depth but also at the level of the costal cartilages [18]. Zhang et al. (2018) showed that PET/CT has an 81.5%, sensitivity for detecting the infection of the costal cartilages, with a 99.8% specificity, and a 98.6% accuracy [19]. Nevertheless, even with this advanced imaging technique, around 20% of the costal infections are missed, which can lead to infection relapse and life-threatening complications.

Although the past years have seen an increased number of publications concerning DSWI, sternal debridement has not been a focus of research so far, with most studies concentrating on the epidemiology, diagnosis, NPWT and flap coverage [20]. The sternal debridement is usually described as the removal of the wires, necrotic soft tissues and bone, without mentioning how far the bone removal should go[15, 20]. The resection of necrotic bony tissue is usually surgically performed with a bone rongeur, removing the bone piece by piece. In the case of sternectomy, this classical procedure theoretically insures on one hand more safety in not injuring the underlying structures (pleura, lungs, remaining internal mammary artery, pericardium, etc.) while on the other hand not guaranteeing the radical sternal removal, especially of the dorsal cortical bone. Furthermore, during resection, there is uncontrolled bleeding from the cancellous bone. The newly described en bloc resection of the hemisternum or the whole sternum if bony healed, ensures a radical and swift sternal removal while avoiding inadvertent bone bleeding. The technique is nevertheless technically more challenging, as the sternum must be released from the underlying structures. The postoperative scaring complicates this surgical step, especially in late DSWI. Altogether, our study proved that both techniques are safe and similar in terms of operative time, intraoperative and postoperative complications and patient survival. As all resected specimens showed histopathological signs of osteomyelitis and no major intraoperative incidents were recorded with both techniques, we conclude that the radical sternectomy down to the ribs and clavicles should be performoseed to secure infection control. Especially patients with extensive clinical necrosis of the sternal bone, those with multiple infection relapses and those who have been hospitalized and wish a fast and relapse-free treatment, will benefit from the radical sternectomy.

The long-term results confirmed furthermore, that both sternectomy techniques ensure long-term infection control with limited relapses at the level of the remaining rib and clavicula stumps, which can be controlled with limited surgical interventions. The multivariate analysis showed that only ASA 4 patients were more prone to develop a fistula, which indicates that the biological status of the patient impeded the complete eradication of the infection. Although the muscle flap coverage enhances the treatment of soft tissue infections, the retaining of necrotic bone could lead to persistent infection [13] with devastating consequences to the patient.

After cardiac surgery, patients usually require the intake of antiplatelet and anticoagulant drugs [21, 22]. The occurrence of a DSWI complicates the treatment of these patients and requires additional surgeries. While some surgeons would tend to pause the antiplatelet agents and reduce the heparin products to prophylactic doses, this could lead to serious thrombotic complications and death. In our study, we could prove that the continuation of these drugs does not significantly complicate the course of treatment. Obesity showed on the other hand to increase the risk of bleeding in overweight patients ongoing sternectomy.

The 9.3% mortality in this cohort was comparable to the literature (10.7% [2]). None of the surgical-related factors has proven to influence patient survival. Although in our cohort there were only 20 women, they appeared to have a worse prognosis than men. This result should be nevertheless carefully interpreted due to the reduced number of patients (intermediate effect size (d = 0.55) and the statistical power 0.52) and the fact that some deaths were not related to the DSWI and its treatment (for example brain metastases or COVID 19 infection). Patients who had to be revised due to bleeding also tended to have a higher risk of death, whereas a larger cohort would need to confirm this result. Relevant bleeding favors bacterial translocation and subsequent sepsis, which seriously complicates the treatment. Considering all the factors which lead to bleeding and increased transfusion, which could finally worsen the prognosis, we recommend considering preventive measures, especially in overweight and ASA 4 patients, like preoperative transfusion to increase the blood hemoglobin, perioperatively reducing the anticoagulation to decrease the PTT, administration of antifibrinolytic agents or postponing the beginning of NPWT for one day. We advise against pausing antiplatelet agents, as this might increase thrombotic events and worsen the prognosis.

## Conclusion

We hereby propose a standard of treatment for DSWI which aims at reducing hospitalization, mortality and chronic infection relapses. Radically removing the infected sternal bone when necessary, ensures a swift and safe infection treatment with little long-term recurrences. The newly described radical en bloc sternectomy technique is a safe treatment of DSWI in appropriate cases and showed a tendency to reduce hospital stay. Further studies with larger patients samples are required to confirm these findings.

### Supplementary Information

**Additional file 1.** **Supplementary Table S1:** Logistic regression for factors predicting significant bleeding after sternal resection. ASA, American Society of Anesthesiologist´s physical status; LIMA, use of the left internal mammary artery as bypass craft. Multivariable adjustment had no effect on the result, leaving obesity as the only risk factor. **Supplementary Table S2:** Effect size, power and sample size calculations for significant outcomes. Sample size is calculated as cases per group for a test power of 0.8.

**Additional file 2: Figure S1.** Deep sternal wound with sternal dehiscence, broken wires and fractured bone. **Figure S2.** Separation of the hemisternum from the dorsal periosteum and mediastinal tissues. **Figure S3.** En bloc Resection of the hemisternum using the Ruskin Liston bone cutting forceps. **Figure S4.** Opening of the left sternoclavicular joint. **Figure S5.** Resection of the united sternum in one piece

**Additional file 3: Video S1.** Radical en bloc sternectomy on the left side. Hemisternum specimens after resection.

## Data Availability

The datasets used and/or analyzed during the current study are available from the corresponding author on reasonable request.

## Abbreviations

ASA: American Society of Anesthesiologists Classification
BMI: Body mass index
CABG: Coronary artery bypass graft
DSWI: Deep sternal wound infection
EBSR: En bloc sternal resection
LDMF: Latissimus dorsi musculocutaneous flap
LIMA: Coronary bypass using the left internal mammary artery
NPWT: Negative wound pressure therapy
PET/CT: Positron emission tomography/computed tomography
PTT: Partial thromboplastin time
PMSR: Piecemeal sternal resection
RCT: Randomized controlled study
SO: Sternal osteomyelitis

## References

[1] Kaul P (2017). Sternal reconstruction after post-sternotomy mediastinitis. J Cardiothorac Surg.

[2] Sears ED, Wu L, Waljee JF, Momoh AO, Zhong L, Chung KC (2016). The impact of deep sternal wound infection on mortality and resource utilization: a population-based study. World J Surg.

[3] Damiani G, Pinnarelli L, Sommella L, Tocco MP, Marvulli M, Magrini P, Ricciardi W (2011). Vacuum-assisted closure therapy for patients with infected sternal wounds: a meta-analysis of current evidence. J Plast Reconstr Aesthet Surg.

[4] Lepelletier D, Bourigault C, Roussel JC, Lasserre C, Leclere B, Corvec S (2013). Epidemiology and prevention of surgical site infections after cardiac surgery. Med Mal Infect.

[5] Morisaki A, Hosono M, Murakami T, Sakaguchi M, Suehiro Y, Nishimura S (2016). Effect of negative pressure wound therapy followed by tissue flaps for deep sternal wound infection after cardiovascular surgery: propensity score matching analysis. Interact Cardiovasc Thorac Surg.

[6] Singh K, Anderson E, Harper JG (2011). Overview and management of sternal wound infection. Semin Plast Surg.

[7] Bota O, Josten C, Borger MA, Spindler N, Langer S (2018). A standardized musculocutaneous flap for the coverage of deep sternal wounds after cardiac surgery. Ann Thorac Surg.

[8] Horan TC, Andrus M, Dudeck MA (2008). CDC/NHSN surveillance definition of health care-associated infection and criteria for specific types of infections in the acute care setting. Am J Infect Control.

[9] Lakens D (2017). Equivalence tests: a practical primer for t tests, correlations, and meta-analyses. Soc Psychol Personal Sci.

[10] Package ‘pwr’; 2018.

[11] Team RC. R: A language and environment for statistical computing; 2018 2018.

[12] Organization WH (2000). Obesity: Preventing and managing the global epidemic; report of a WHO Consultation; [1999, Geneva].

[13] Rao N, Ziran BH, Lipsky BA (2011). Treating osteomyelitis: antibiotics and surgery. Plast Reconstr Surg.

[14] Hämäläinen E, Laurikka J, Huhtala H, Järvinen O (2021). Vacuum assistance therapy as compared to early reconstructive treatment in deep sternal wound infection. Scand J Surg.

[15] Yousafzai SM, Ugurlucan M, Awan A, Canver CC (2022). Cost and clinical effectiveness of aggressive surgical debridement and delayed primary closure of infected cardiac surgical wounds. J Wound Care.

[16] Blüher M, Brandt D, Lankiewicz J, Mallow PJ, Saunders R (2020). Economic analysis of the European healthcare burden of sternal-wound infections following coronary artery bypass graft. Front Public Health.

[17] Sears ED, Momoh AO, Chung KC, Lu Y-T, Zhong L, Waljee JF (2017). A national study of the impact of delayed flap timing for treatment of patients with deep sternal wound infection. Plast Reconstr Surg.

[18] Liu S, Zhang J, Yin H, Pang L, Wu B, Shi H (2020). The value of 18 F-FDG PET/CT in diagnosing and localising deep sternal wound infection to guide surgical debridement. Int Wound J.

[19] Zhang R, Feng Z, Zhang Y, Tan H, Wang J, Qi F (2018). Diagnostic value of fluorine-18 deoxyglucose positron emission tomography/computed tomography in deep sternal wound infection. J Plast Reconstr Aesthet Surg.

[20] Hever P, Singh P, Eiben I, Eiben P, Nikkhah D (2021). The management of deep sternal wound infection: literature review and reconstructive algorithm. JPRAS Open.

[21] Weiss A, Brose S, Ploetze K, Matschke K (2013). Half-dose enoxaparin vs. full-dose enoxaparin for postoperative bridging therapy in patients after cardiac surgery: which dose regimen should be preferred?. Clin Hemorheol Microcirc.

[22] Agarwal N, Mahmoud AN, Patel NK, Jain A, Garg J, Mojadidi MK (2018). Meta-analysis of aspirin versus dual antiplatelet therapy following coronary artery bypass grafting. Am J Cardiol.

