# Supplementary material for: The impact of two radical sternectomy surgical techniques on the outcome of deep sternal wound infections
Source: J Cardiothorac Surg. 2024 Jan 24;19:25. doi: 10.1186/s13019-024-02491-7 (PMC10809468; doi:10.1186/s13019-024-02491-7)
Supplement: Supplementary file 1 — Additional file 1. Supplementary Table S1: Logistic regression for factors predicting significant bleeding after sternal resection. ASA, American Society of Anesthesiologist´s physical status; LIMA, use of the left internal mammary artery as bypass craft. Multivariable adjustment had no effect on the result, leaving obesity as the only risk factor. Supplementary Table S2: Effect size, power and sample size calculations for significant outcomes. Sample size is calculated as cases per group for a test power of 0.8. [file 13019_2024_2491_MOESM1_ESM.docx]

| *Predictors* | *Odds Ratio* | *CI* | *p* |
| --- | --- | --- | --- |
| Univariable regression | | | |
| Resection technique | 1.03 | 0.22 – 7.45 | 0.968 |
| Platelet inhibition |  |  |  |
| single | 1.26 | 0.20 – 24.80 | 0.835 |
| double | 0.00 | n.a. | 0.993 |
| anticoagulation | 1.10 | 0.25 – 5.67 | 0.902 |
| partial thromboplastin time | 1.12 | 1.01 – 1.29 | **0.042** |
| prothrombine time | 0.99 | 0.96 – 1.04 | 0.668 |
| ASA Class 4 | 3.30 | 0.61 – 15.38 | 0.133 |
| Age | 0.92 | 0.83 – 1.02 | 0.109 |
| Femal gender | 0.00 | n.a. | 0.994 |
| Obesity | 10.1 | 1.68 – 192.9 | **0.035** |
| LIMA graft | 1.25 | 0.27 – 9.00 | 0.790 |
| Preoperative hemoglobine | 0.79 | 0.30 – 1.64 | 0.574 |
| Supplementary Table 1: Logistic regression for factors predicting significant bleeding after sternal resection. ASA, American Society of Anesthesiologist´s physical status; LIMA, use of the left internal mammary artery as bypass craft. Multivariable adjustment had no effect on the result, leaving obesity as the only risk factor. | | | |

| Factor | Effect | Effect size | Power | Sample size |
| --- | --- | --- | --- | --- |
| obesity | higher risk of reintervention for bleeding in obese patients | 0.58 | 0.82 | 37 |
| insuline therapy | higher mortality in patients chronically treated with insulin | 0.48 | 0.65 | 54 |
| gender | higher mortality in women | 0.43 | 0.52 | 67 |
| bleeding | higher mortality in men with bleeding requiring reinterventiion | 0.48 | 0.36 | 53 |
| ASA physical status | higher likelihood of postoperative fistula in ASA 4 patients | 0.55 | 0.46 | 42 |
| Supplementary Table 2: Effect size, power and sample size calculations for significant outcomes. Sample size is calculated as cases per group for a test power of 0.8. | | | | |
