# Supplementary figures and images for: The impact of two radical sternectomy surgical techniques on the outcome of deep sternal wound infections
Source: J Cardiothorac Surg. 2024 Jan 24;19:25. doi: 10.1186/s13019-024-02491-7 (PMC10809468; doi:10.1186/s13019-024-02491-7)

## Slide 1
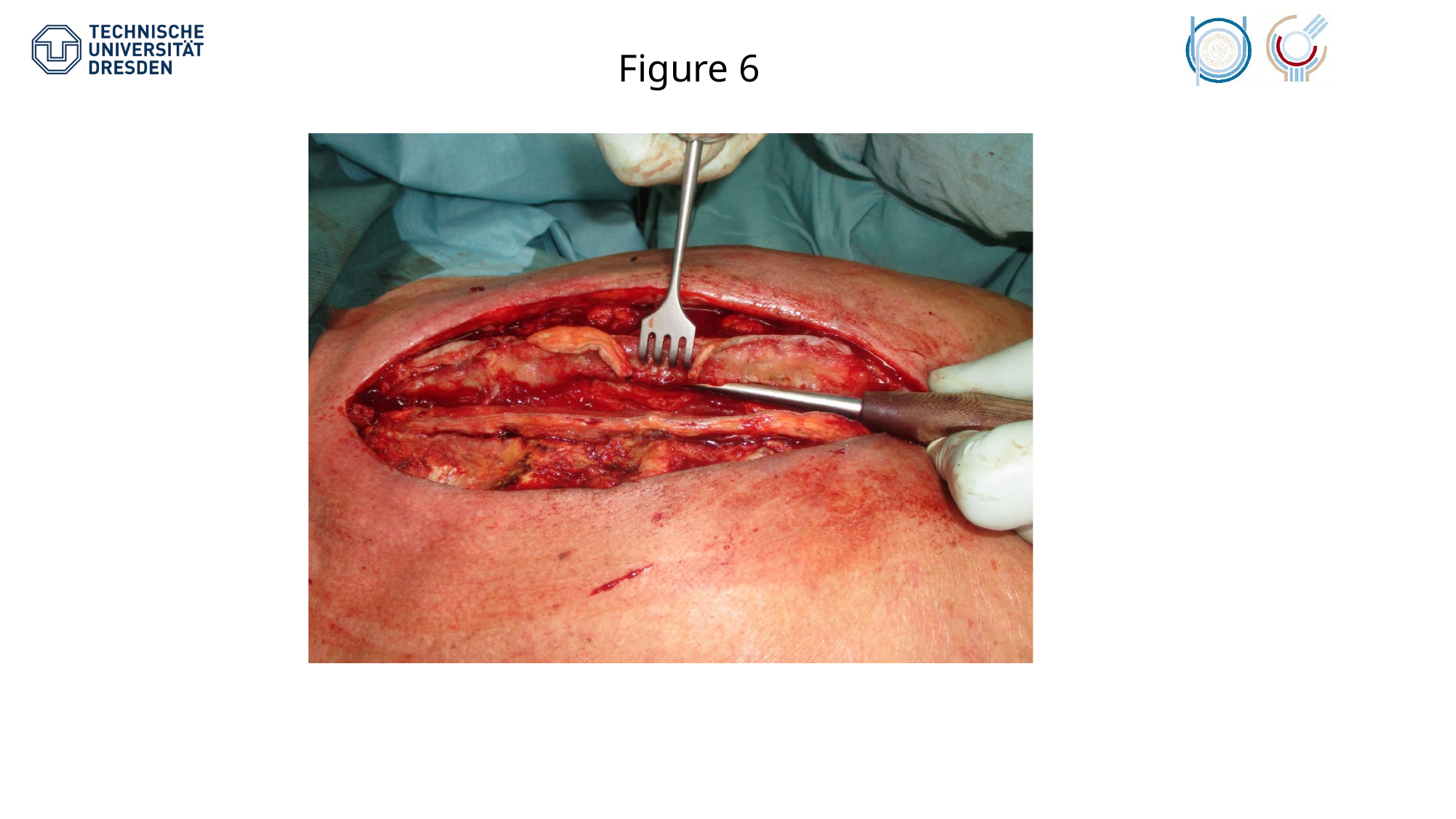

Figure 6

## Slide 2
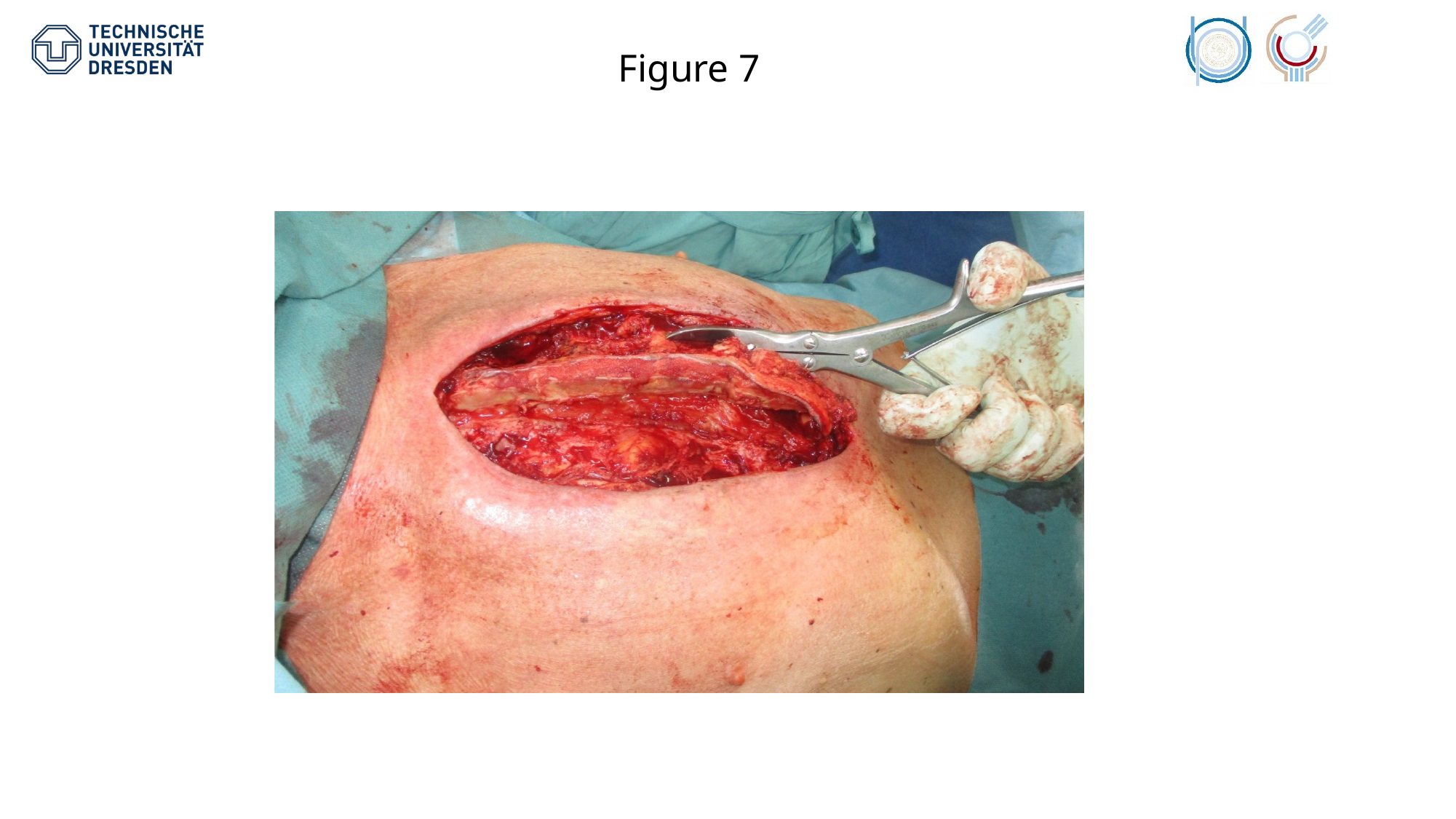

Figure 7

## Slide 3
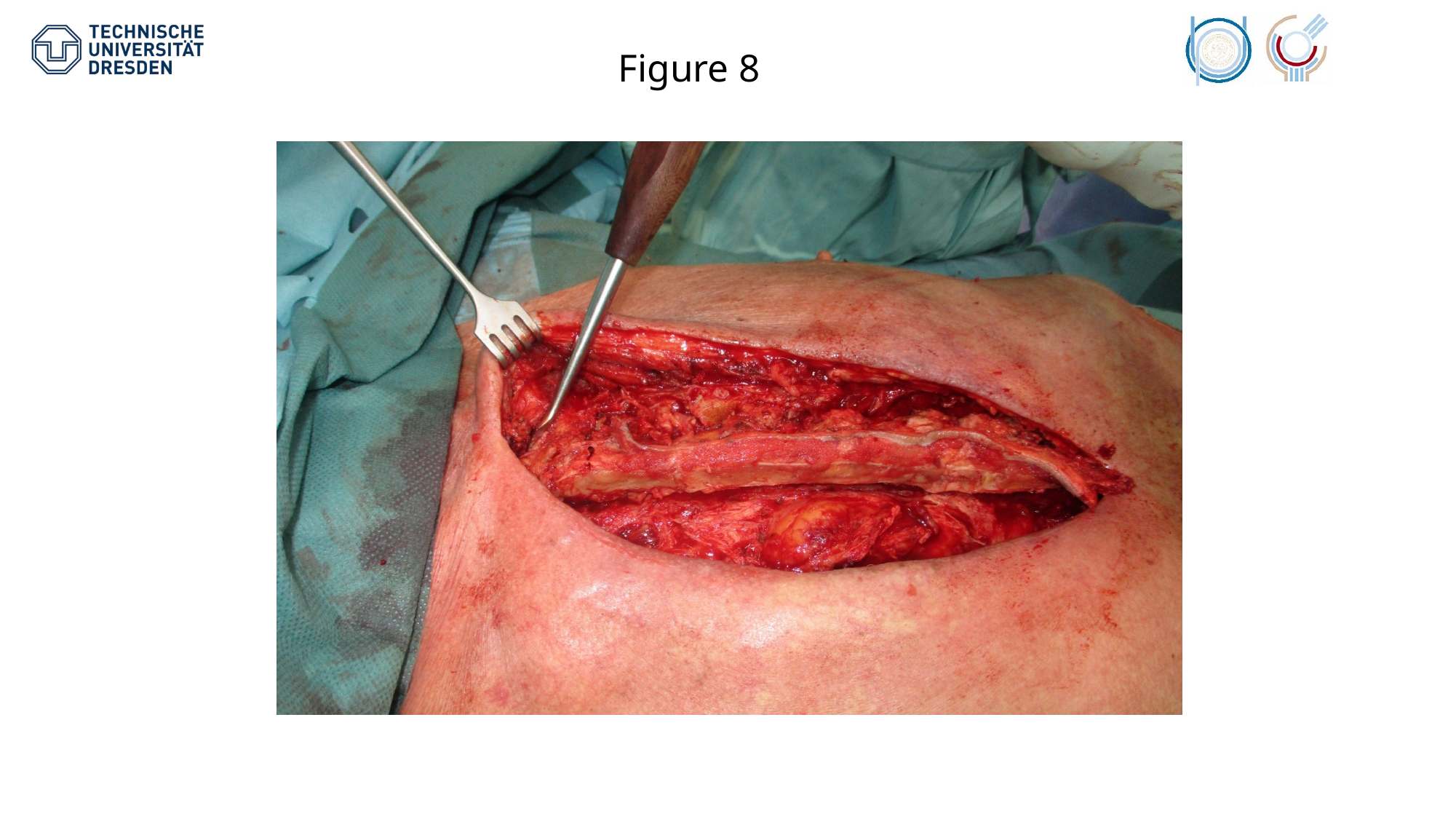

Figure 8

## Slide 4
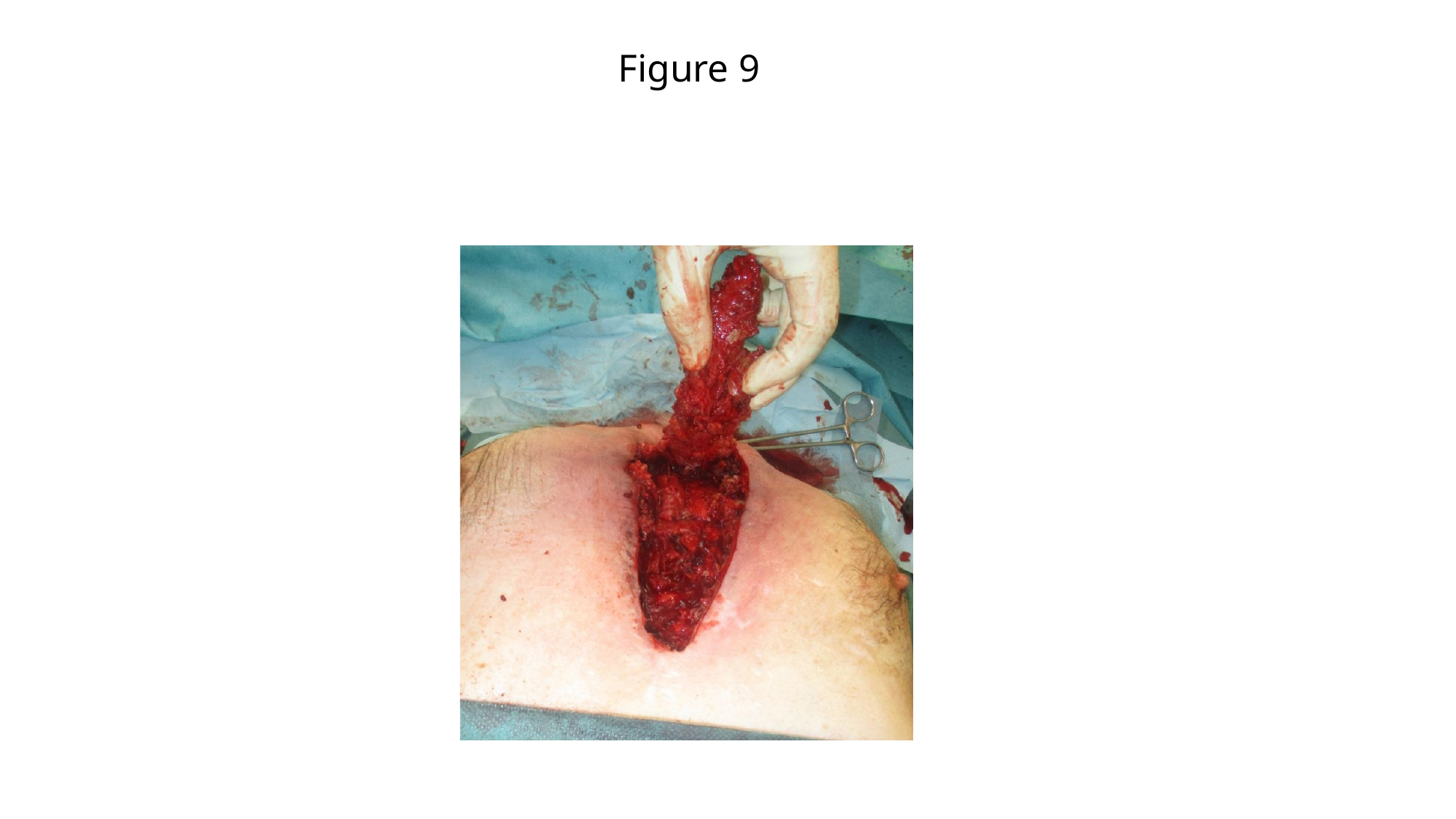

Figure 9

## Slide 5
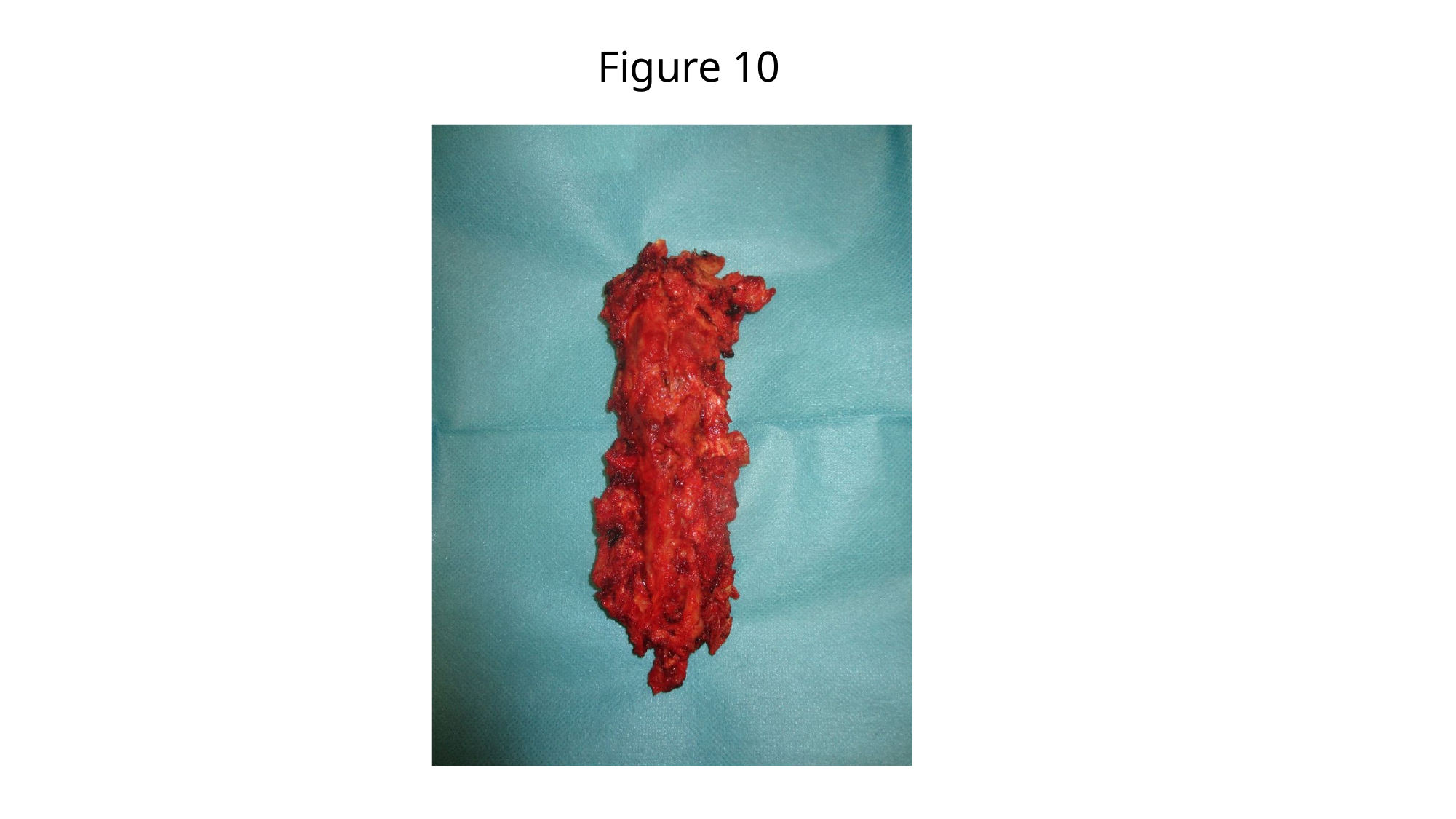

Figure 10

Supplement: Supplementary file 2 — Additional file 2: Figure S1. Deep sternal wound with sternal dehiscence, broken wires and fractured bone. Figure S2. Separation of the hemisternum from the dorsal periosteum and mediastinal tissues. Figure S3. En bloc Resection of the hemisternum using the Ruskin Liston bone cutting forceps. Figure S4. Opening of the left sternoclavicular joint. Figure S5. Resection of the united sternum in one piece [file 13019_2024_2491_MOESM2_ESM.pptx]
